# Supplementary material for: Photoinduced semiconductor-metal transition in ultrathin troilite FeS nanosheets to trigger efficient hydrogen evolution
Source: Nat Commun. 2019 Jan 23;10:399. doi: 10.1038/s41467-019-08358-z (PMC6344495; doi:10.1038/s41467-019-08358-z)
Supplement: Supplementary file 1 — Supplementary Information [file 41467_2019_8358_MOESM1_ESM.pdf]

Supplementary Information for Manuscript

Photoinduced Semiconductor-Metal Transition in Ultrathin Troilite FeS Nanosheets to Trigger Efficient Hydrogen Evolution

Zhou et al.

Supplementary Figures

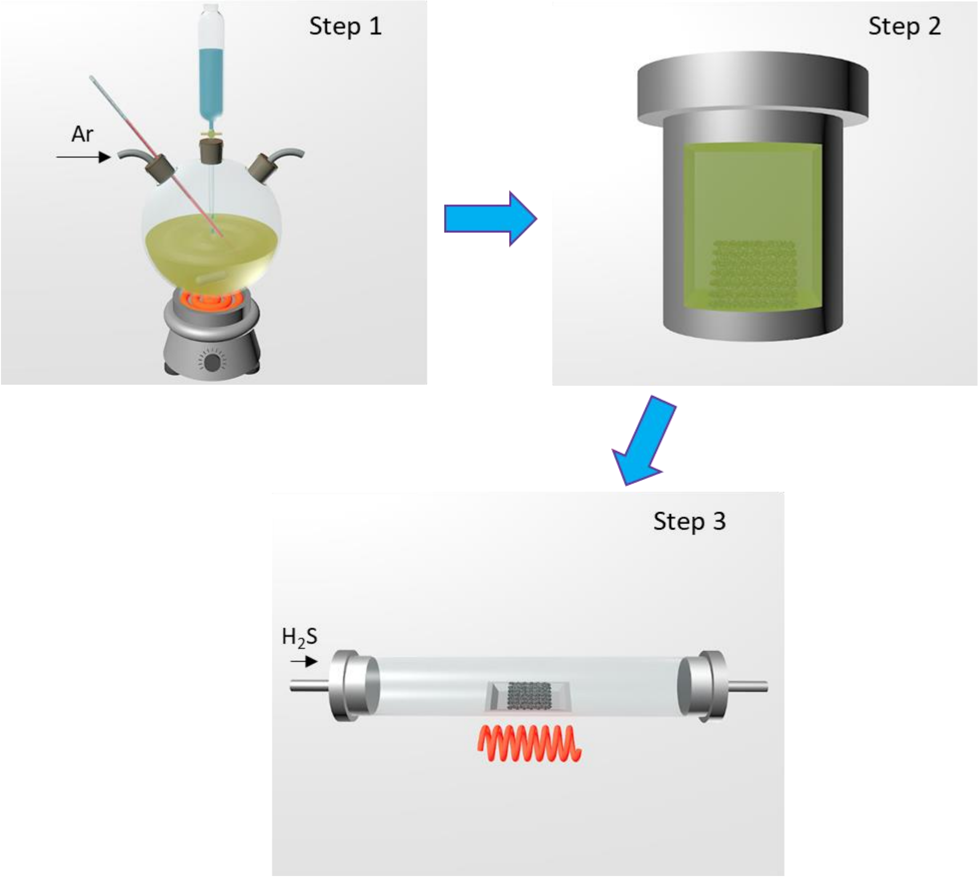

Supplementary Figure 1. The schematic representation of preparation route for FeS nanosheets.

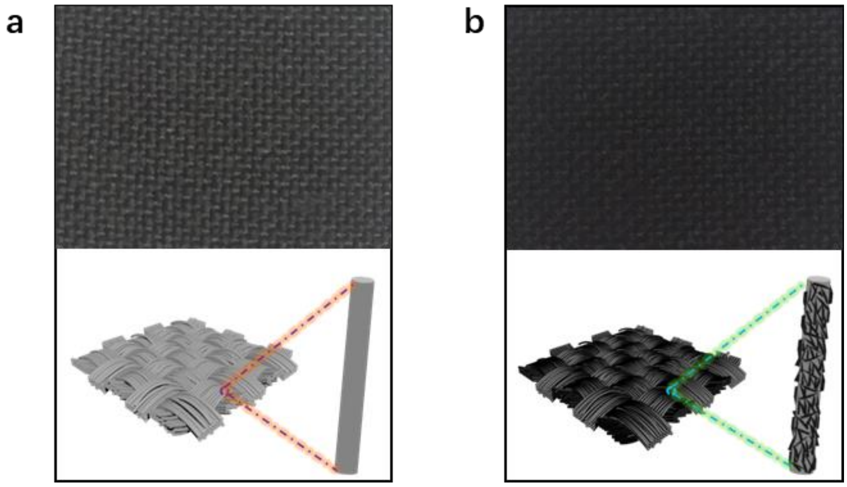

Supplementary Figure 2. Digital photographs (top) and schematic illustration (bottom) of CFC (a) and FeS/CFC (b) electrodes.

The analysis about digital photographs and schematic illustration of CFC (a) and FeS/CFC (b) electrodes shows that the amount of FeS deposited on CFC can be obtained by the quantity difference between CFC [m(CFC)=34.8 mg] and FeS/CFC [m(FeS/CFC)=35.2 mg] . The surface area of CFC is about S=2.5 cm^2. Then amount of FeS can be calculated to be:

$$\emptyset = \frac{\mathrm{m(FeS/CFC)-m(CFC)}}{\mathrm{S}} = 0.16\, mg/cm^2 \quad (1)$$

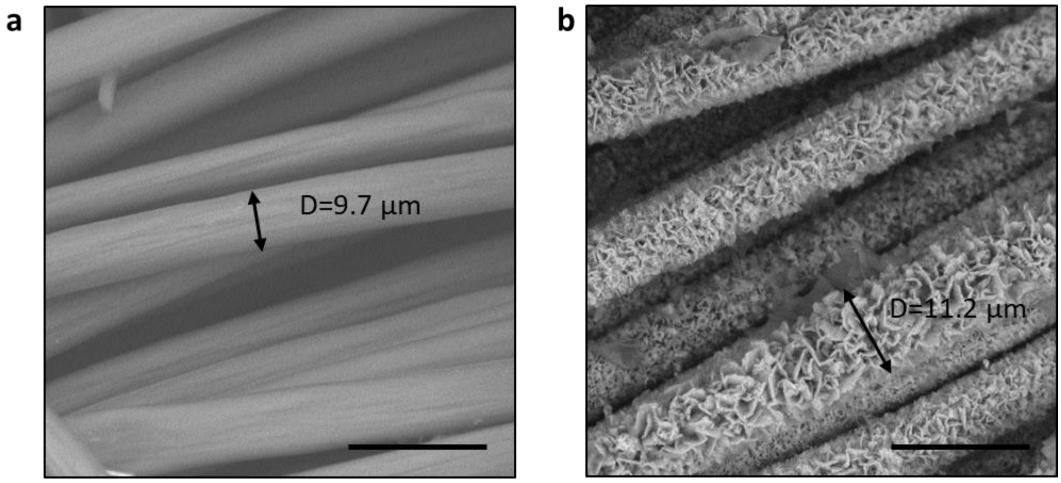

Supplementary Figure 3. The SEM images of bare CFC (a) and CFC decorated with FeS nanosheets (b). (Scale bar, 20 µm)

The densely loaded vertical FeS nanosheets are averagely of 700-800 nm in height.

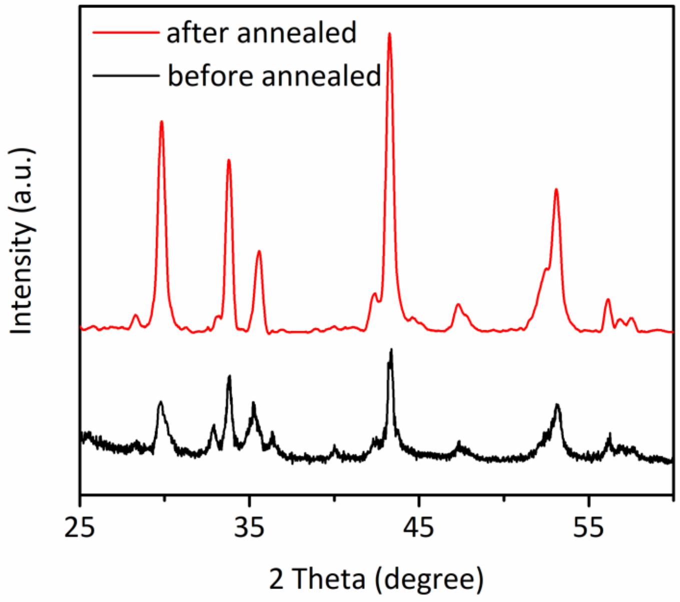

Supplementary Figure 4. XRD patterns of FeS nanosheets before and after thermal treatment in H_2S/Ar atmosphere.

The crystallinity and purity of FeS is improved by the thermal treatment, respectively. As seen in Supplementary Figure 4, some weak XRD peaks originating from iron deficiencies or other iron-sulfur compounds were removed by subsequent thermal treatment in H_2S/Ar atmosphere. EDS analysis confirms that the Fe/S ratio is enhanced from 0.83:1 (before annealing) to around 1:1 (after annealing) via the thermal treatment.

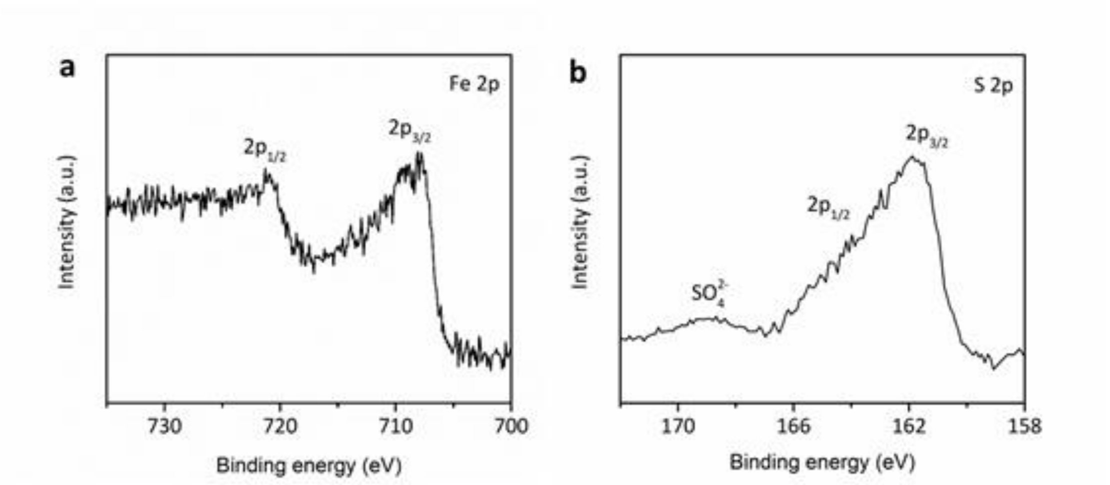

Supplementary Figure 5. High-resolution XPS spectra in (a) Fe 2p and (b) S 2p regions of FeS nanosheets before thermal treatment.

The fine XPS spectra of Fe 2p (Supplementary Figure 5a) and S 2p (Supplementary Figure 5b) indicate that the binding energies of Fe 2p1/2, Fe 2p3/2, S 2p1/2 and S 2p3/2 are located at 721.3, 708.1, 163.8 and 161.9 eV, respectively. In this unannealed sample, a weak XPS peak at 169.5 eV originating from surface sulfate moieties (SO_4^{2-}) can be observed due to the polyol synthesis progress of metal sulfides. In comparison with the annealed sample in Supplementary Figure 20, this weak XPS peak from SO_4^{2-} can be removed. It is important to note that the characteristic peaks of Fe 2p shift toward higher binding energy after thermal treatment in H_2S atmosphere, which can be ascribed to the decreased electron density induced by disappearance of iron deficiencies and sulfate moieties. These results can also be confirmed by XRD in Supplementary Figure 4.

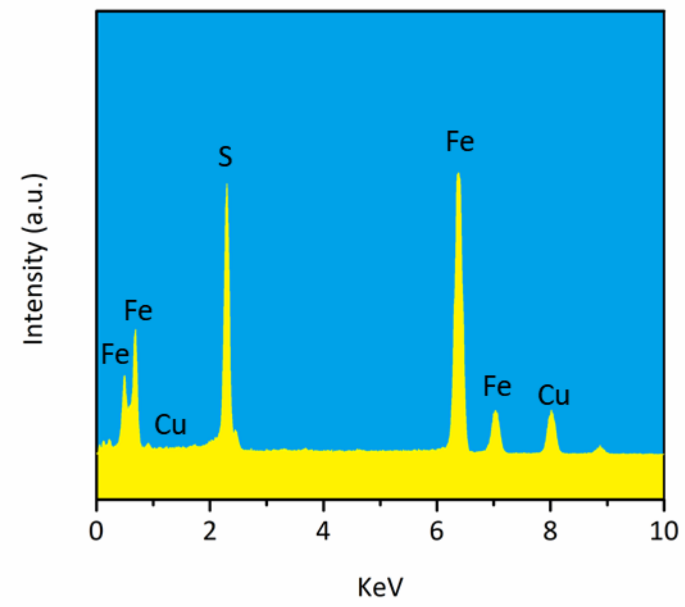

Supplementary Figure 6. EDS spectrum of the FeS nanosheet.

No noticeable impurities are introduced unintentionally in the preparation process. The detected Cu element is derived from the Cu mesh braces. The nanosheet is composed mainly of Fe and S atoms at an approximate ratio of 1:1.

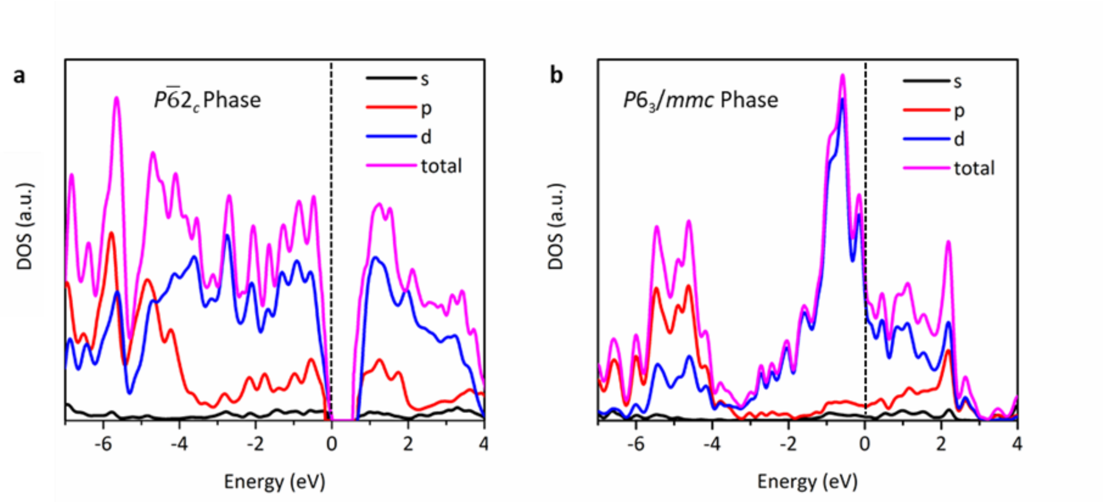

Supplementary Figure 7. Project Density of states (DOS) of $P\bar{6}2_c$ (a) and $P6_3/mmc$ (b) FeS phase. The CBM and VBM near the Fermi level largely originate from the *d* orbit of Fe and the effect of the *p* and *s* orbit of S is very small. In addition, a band gap of ~0.5 eV can be observed in $P\bar{6}2_c$ phase, displaying an obvious semiconductor feature. On the contrary, in $P6_3/mmc$ structure, the band gap disappears and the Fermi level is also occupied completely, showing a typical metal feature. This semiconductor-metal transition has an obvious advantage in carrier transfer.

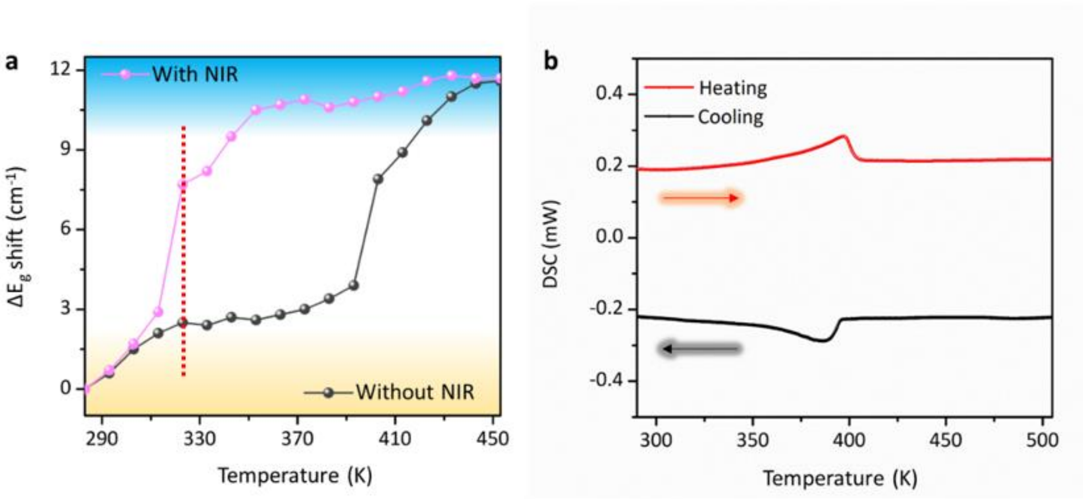

Supplementary Figure 8. Peak shifts of E_g modes at different temperatures observed from *in situ* Raman spectra (a) and differential scanning calorimetry (DSC) curves (b) of the FeS nanosheets.

The peak shifts of E_g modes at different temperatures observed from *in situ* Raman spectra, relative to E_g peak at 285 K, confirms that the E_g modes are sharply down-shifted due to phase transition at 403 K without NIR light radiation. It is generally accepted that the shift of Raman mode corresponds to structural transition. Therefore, compared to that without NIR light irradiation, the sharp shifts of E_g modes induced by NIR light irradiation at initial stage (~ 323 K, marked by red line) make us to determine the transition temperature facilely. Obviously, the transition can occur at 323 K once introducing NIR light radiation (Supplementary Figure 8a). The DSC curves show two peaks at 397 and 386 K in the heating and cooling runs respectively, indicating a reversible structural phase transition between $P\overline{6}2_c$ and $P6_3/mmc$ at ca.397 K (Supplementary Figure 8b).

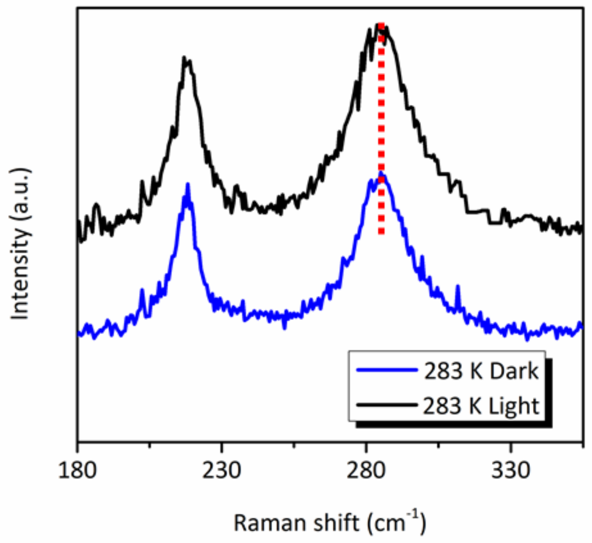

Supplementary Figure 9. ***In situ*** **Raman spectra at 283 K with and without visible light irradiation (532 nm).**

The 532 nm light makes no change in the out-of-plane E_g mode.

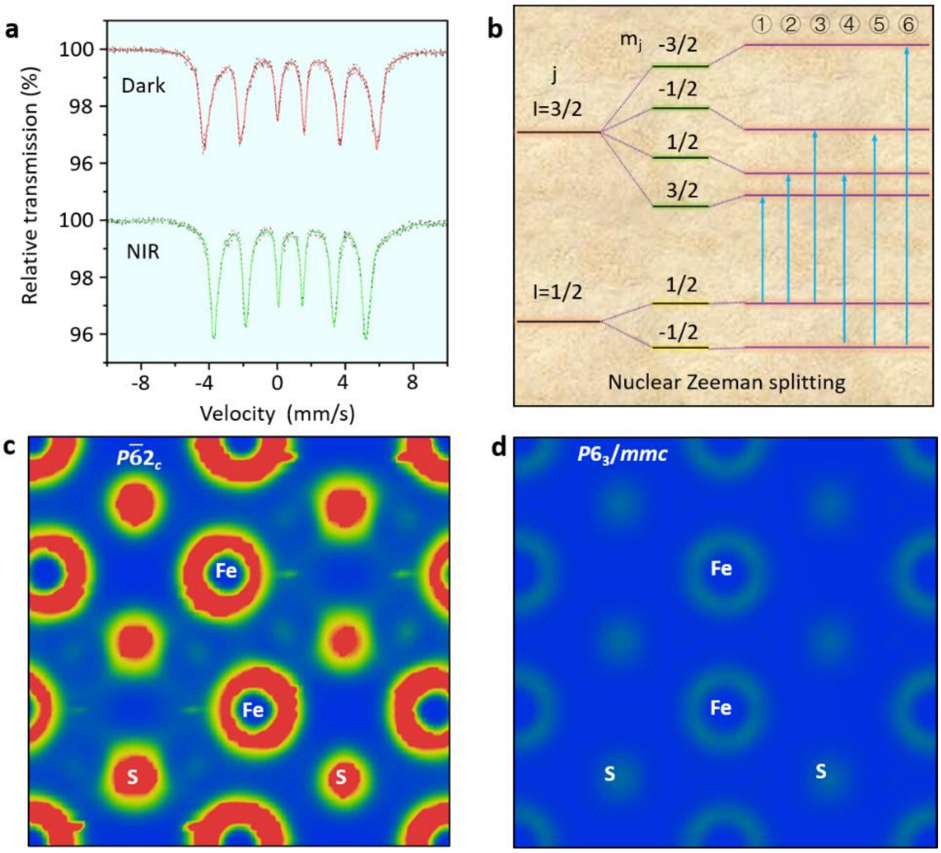

Supplementary Figure 10. The analysis about phase transition by MÖssbauer spectra and DFT. The ^{57}Fe MÖssbauer spectra of FeS nanosheets with and without NIR light irradiation (a). The schematic illustration of nuclear Zeeman splitting (b). The calculated electron localization function of the (100) surface of *$P\bar{6}2_c$* (c) phase and its corresponding surface in *$P6_3/mmc$* (d) phase.

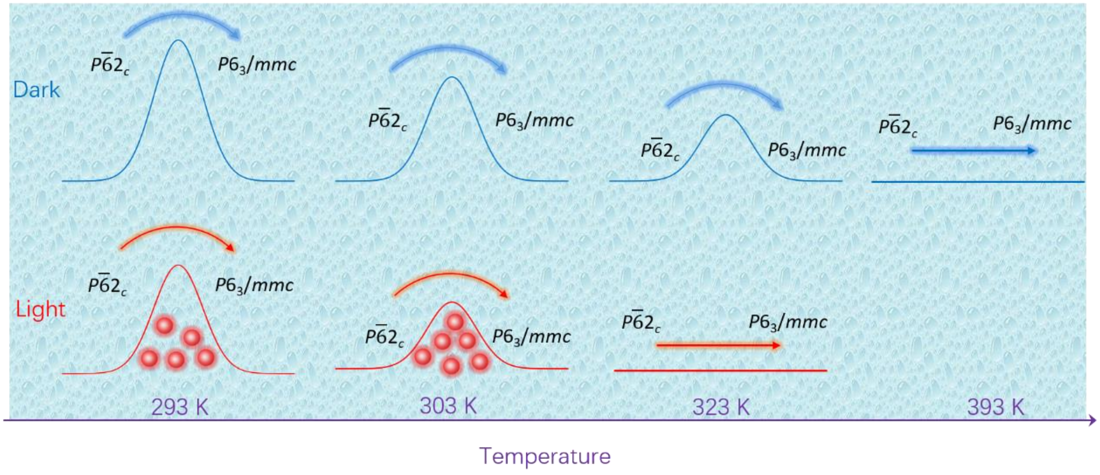

Supplementary Figure 11. Schematic representation for potential barrier versus temperature with and without NIR light irradiation.

In order to more intuitively display the phase transition process, the potential barrier as function of temperature with and without NIR light irradiation are schematically displayed in Supplementary Figure 11. The transition potential barrier from $P\bar{6}2_c$ to $P6_3/mmc$ phase is obviously reduced by accumulating photogenerated carrier at the same temperature, which finally make the phase transition temperature decrease from 393 K to 323 K.

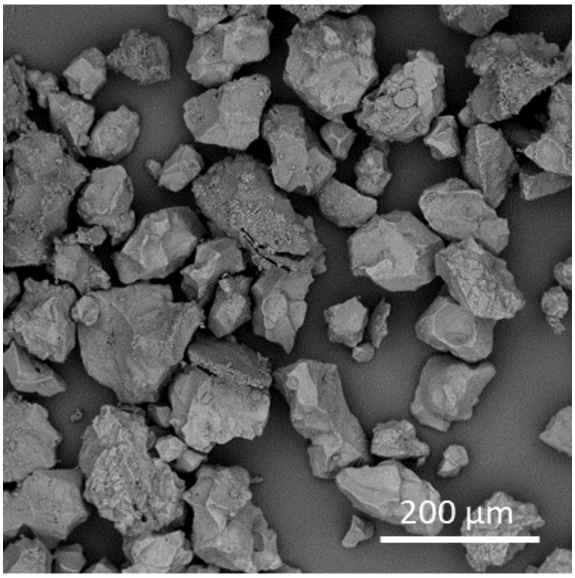

Supplementary Figure 12. SEM image of commercial FeS powders.

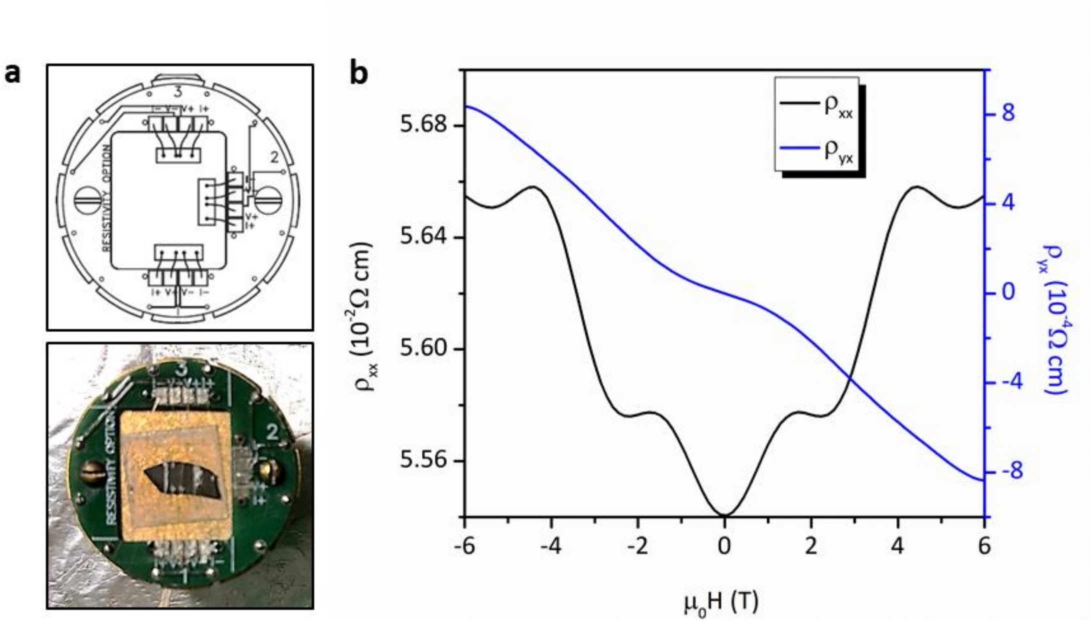

Supplementary Figure 13. Schematic diagram and photo image of sample stage (a) and the longitudinal resistivity and hall resistivity (b).

The real longitudinal resistance (R_{xx}) and hall resistance (R_{yx}) signals were obtained from the corrected equations as follow:

$$R_{xx}(H) = (R'_{xx}(H) + R'_{xx}(-H))/2 \tag{2}$$

$$R_{yx}(H) = (R'_{yx}(H) - R'_{yx}(-H))/2 \tag{3}$$

where the R'_{xx} and R'_{xx} were the original signal. We further used the equation $[\rho = (R \cdot S)/L]$ to achieve the longitudinal resistivity and hall resistivity as shown in Figure S13b, where the R, S and L were the resistance, cross-sectional area and transmission distance, respectively. The resistivity can be converted to the conductivity tensor using the following functions:

$$\sigma_{xx} = \frac{\rho_{xx}}{\rho_{xx}^2 + \rho_{yx}^2} \tag{4}$$

$$\sigma_{yx} = \frac{\rho_{yx}}{\rho_{xx}^2 + \rho_{yx}^2} \tag{5}$$

In the quasi-classical approximation, the longitudinal/lateral conductivity contributed

by a single energy band (assumed as holes) and the electrical parameters as the carrier concentration (n) and mobility (μ) under magnetic field (H) have the following relations:

$$\sigma_{xx} = \frac{en\mu}{1+(\mu H)^2} \tag{6}$$

$$\sigma_{yx} = \frac{en\mu^2 H}{1+(\mu H)^2} \tag{7}$$

$$\frac{\sigma_{yx}}{\sigma_{xx}} = \frac{\rho_{yx}}{\rho_{xx}} = \mu H \tag{8}$$

According to the above the equations, the mobility can be obtained from the following function and the mobility of FeS nanosheets is estimated to be 11.217 $cm^2\ V^{-1}\ s^{-1}$.

$$\lim_{H\to 0} \mu = \lim_{H\to 0} \frac{\frac{d\rho_{yx}}{dH}}{\rho_{xx}} \tag{9}$$

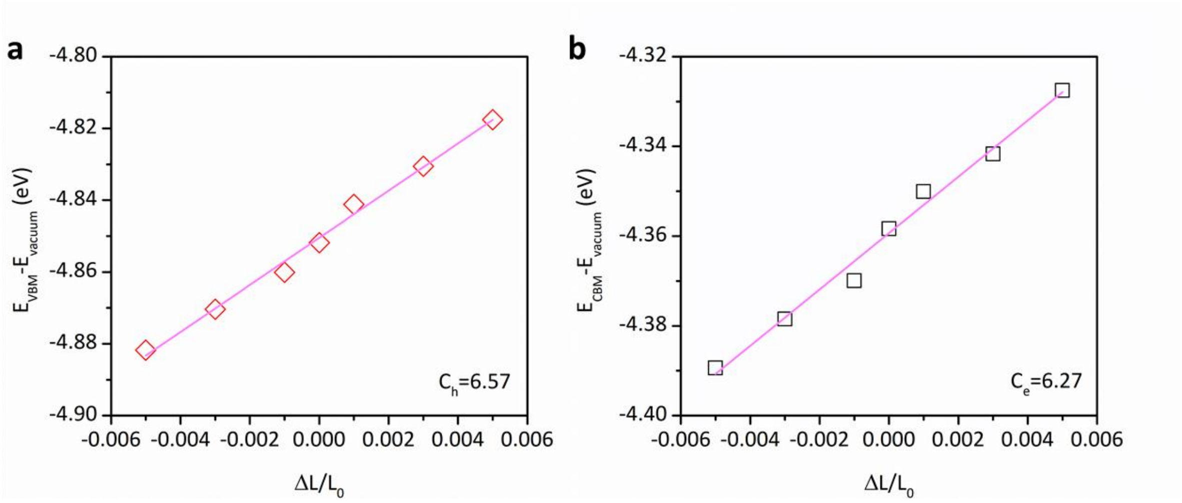

Supplementary Figure 14. Band energy of the CBM (a) and VBM (b) of $P\overline{6}2_c$ phase FeS with respect to the vacuum energy as a function of lattice dilation.

The carrier mobility can be calculated from the equation:

$$\mu_D = \frac{e\hbar^3 C_D}{k_B T m_e^* m_d (E_1^i)^2} \quad (10)$$

Where m_e^* is the effective mass in the transport direction and m_d is the average effective mass determined by its anisotropy. The term E_1 represents the deformation potential constant of the valence-band minimum for the hole or conduction-band maximum for electron along the transport direction, defined by:

$$E_1^i = \Delta V_i / (\Delta l / l_0) \quad (11)$$

Here ΔV_i is the energy change of the ith band under proper cell compression and dilatation (calculated using a step of 0.5%), l_0 is the lattice constant in the transport direction and Δl is the deformation of l_0. The C_D and T is the elastic modulus and calculated temperature, respectively. All structural properties in our calculation of carrier mobility were obtained from DFT calculation. According above theoretical derivation, the mobility of electron and hole is 15.76 and 25.41 $cm^2 V^{-1} s^{-1}$, close to our experimental measurement.

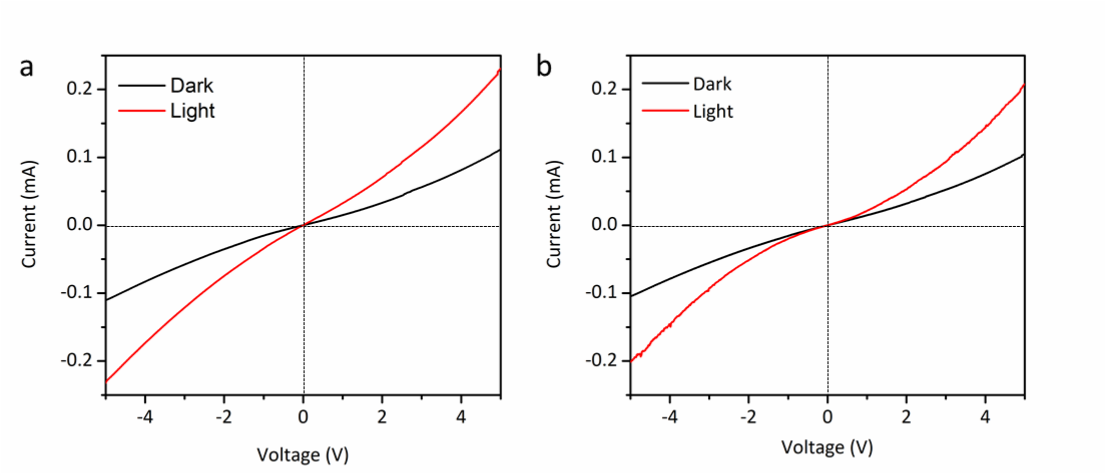

Supplementary Figure 15. *I-V* curves of the photodetector with and without NIR light (1064 nm) illumination at 336.9 K (a) and 328.4 K (b).

The current with and without light at 336.9 K has no obvious change compared with that at 328.4 K.

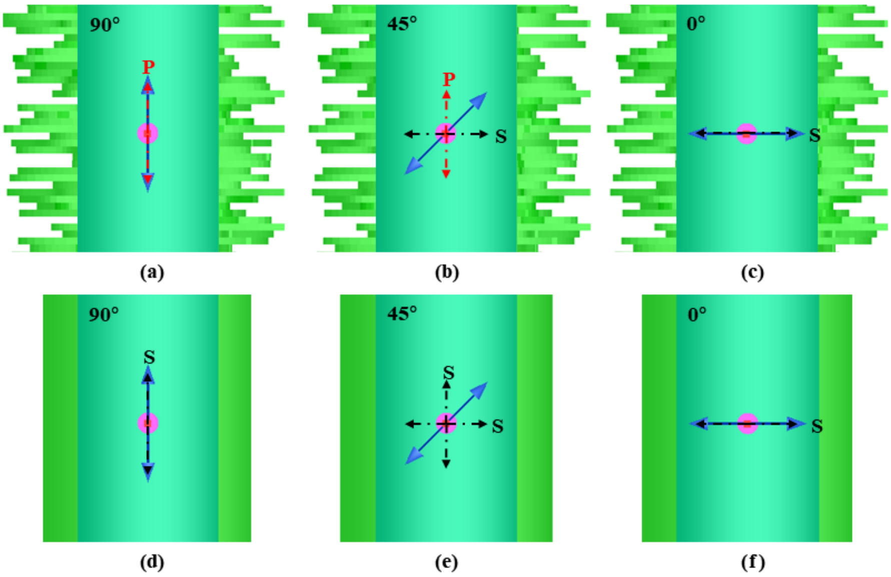

Supplementary Figure 16. Simulation of polarization absorption characteristics based on Finite-Difference Time-Domain (FDTD) method.

The models of FeS nanosheets perpendicular to the main axis of the carbon fiber (top) and accumulated (core–shell) structure (bottom) are displayed in Supplementary Figure 16 a-c and Supplementary Figure 16 d-f, respectively. Besides, the incident plane waves of different polarization orientations, such as 0°, 45° and 90°, are given to study the effects of polarization state of light. The middle drawings of top and bottom are just utilized to indicate a common polarization direction, which can be decomposed into P-waves and S-waves based on Jones vector theory. As for the FeS/CFC (top), arbitrarily polarized incident plane waves almost appear as P waves except the horizontal polarization, while they always exhibit as S waves in FeS/CFC with the core–shell structure (bottom). For the nanosheets, the eigenmatrices of the membrane for S-wave and P-wave are

$$M=\begin{bmatrix}\cos\delta & -\frac{i}{\eta_{1m}}\sin\delta \\ -i\eta_{1m}\sin\delta & \cos\eta_{1m}\end{bmatrix},(m=s,p). \tag{12}$$

Where

$$\delta=\frac{2\pi}{\lambda}n_1 h\cos\theta, \tag{13}$$

$$\eta_{1s}=\sqrt{\frac{\varepsilon_0}{\mu_0}}n_1\cos\theta, \tag{14}$$

$$\eta_{1p}=\sqrt{\frac{\varepsilon_0}{\mu_0}}\frac{n_1}{\cos\theta}. \tag{15}$$

θ is the incident angle of light in thin films, n_1 is the refractive index of film, h is the thickness of film, ε_0 and μ_0 are the permittivity and permeability constants respectively. Then, the reflectivity and transmissivity of the film can be obtained below:

$$R=\frac{(\eta_0-\eta_2)^2\cos^2\delta+(\frac{\eta_0\eta_2}{\eta_1}-\eta_1)^2\sin^2\delta}{(\eta_0+\eta_2)^2\cos^2\delta+(\frac{\eta_0\eta_2}{\eta_1}+\eta_1)^2\sin^2\delta}, \tag{16}$$

$$T=\frac{4\eta_0{}^2}{(\eta_0+\eta_2)^2\cos^2\delta+(\frac{\eta_0\eta_2}{\eta_1}+\eta_1)^2\sin^2\delta}. \tag{17}$$

Where η_0 and η_2 are the related parameters of the medium on both sides of the film.

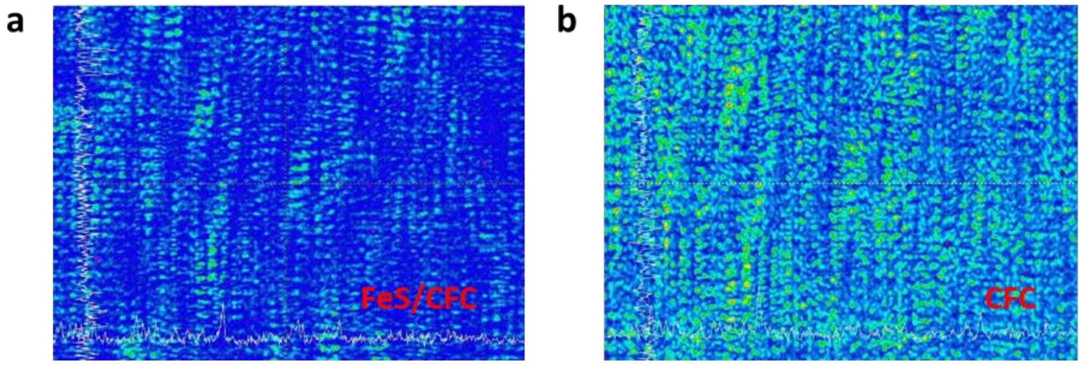

Supplementary Figure 17. CCD imaging of FeS/CFC with vertically oriented nanosheets structure (a) and CFC (b) under the illumination of NIR light.

The measurement system consists of an infrared light source and a charge-coupled device (CCD) camera. The light source and the CCD camera are placed vertically above the line separating the FeS/CFC and CFC to be measured. More weakly reflected light intensity of FeS/CFC than that of CFC proves the excellent absorption ability (I_a=I_{total}-I_r) of FeS/CFC with vertically oriented nanosheets structure.

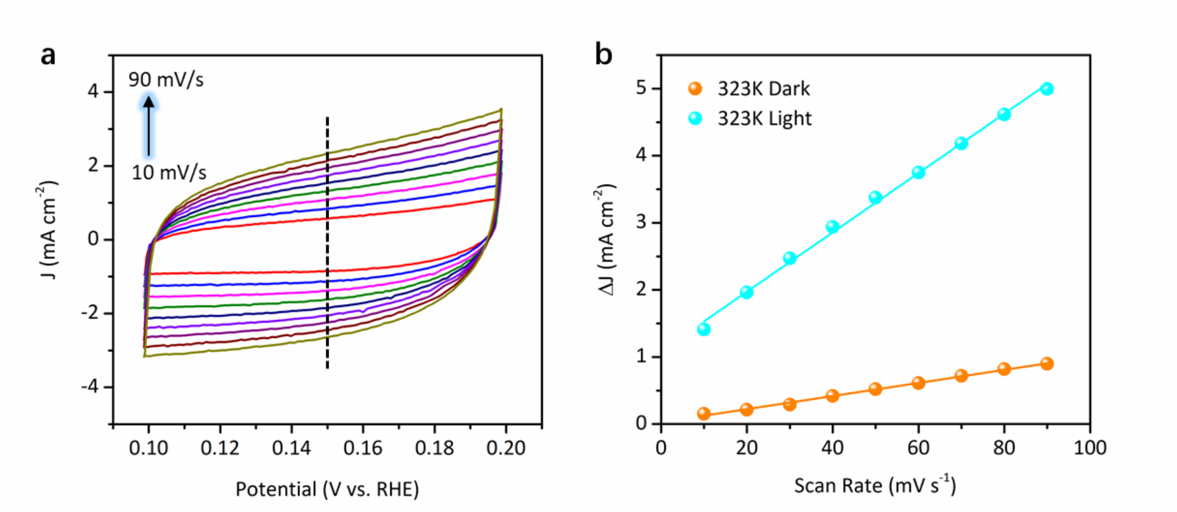

Supplementary Figure 18. (a) FeS nanosheets under NIR light irradiation at 323K with various scan rates. (b) Charging current density differences plotted against scan rates at 0.15 V. The linear slope, equivalent to twice the double-layer capacitance, C_{dl}, was used to represent the ECSA.

To assess the electrochemical active surface area (ECSA), double layer capacitance (C_{dl}) of the catalyst was measured by a simple cyclic voltammetry method (Supplementary Figure 18a) to roughly calculate the value of ECSA. The current density at the selected potential from the regions of no Faradaic processes shows the linear correlation with the scan rate and the slope of the fitting curves is considered as the C_{dl}. In Supplementary Figure 18b, the C_{dl} of $P6_3/mmc$ phase has the ECSA value of 552.5 cm^2, which is about 4.5 times larger than that of $P\overline{6}2_c$ phase (122.5 cm^2), suggesting the enhanced catalytic active sites for HER. In consequence, the increased catalytic active site enabled by phase trasition results in the significant improvement of catalytic performance.

To measure electrochemical double-layer capacitance (C_{dl}), the potential was swept nine times at each scan rate (10, 20, 30, 40, 50, 60, 70, 80, and 90 mV/s) in the scan

range from 0.10 to 0.20 V vs. RHE. Capacitive currents were measured in a potential range where no faradic processes were observed. The measured capacitive currents difference (ΔJ) at 0.15 V vs. RHE was plotted against scan rate and specific capacitance was determined from the slope of the linear fitting. The C_{dl} values for $P\bar{6}2_c$ phase and $P6_3/mmc$ phase are calculated to be 4.9 and 22.1 mF·cm^{-2}, respectively. The specific capacitance is converted into an electrochemical surface area (ECSA) using the specific capacitance value for a flat standard with 1 cm^2 of real surface area. We use the specific capacitance (20–60 μF cm^{-2}) of 40 μF cm^{-2} here to calculate the ECSA[1,2]according to the Eq:

$$\mathrm{ECSA} = \frac{C_{\mathrm{d1}}}{40\,uF/cm^2}\,cm^2_{\mathrm{ECSA}} \tag{18}$$

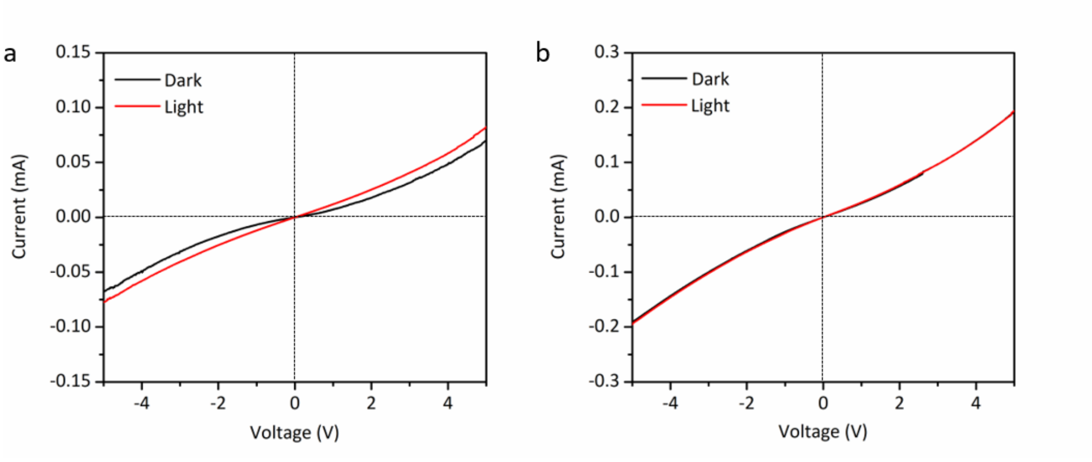

Supplementary Figure 19. *I-V* curves of the photodetector with and without NIR light illumination at 298 K (a) and 424 K (b).

As shown in Supplementary Figure 19a, the *I-V* curves of FeS nanosheets with and without NIR light irradiation has no remarkable differences due to the absence of phase transition at 298 K. There is no photoresponse after the phase transition of FeS nanosheets from $P\bar{6}2_c$ to $P6_3/mmc$ finishes, which further proves the important effect of the phase transition on HER (Supplementary Figure 19b).

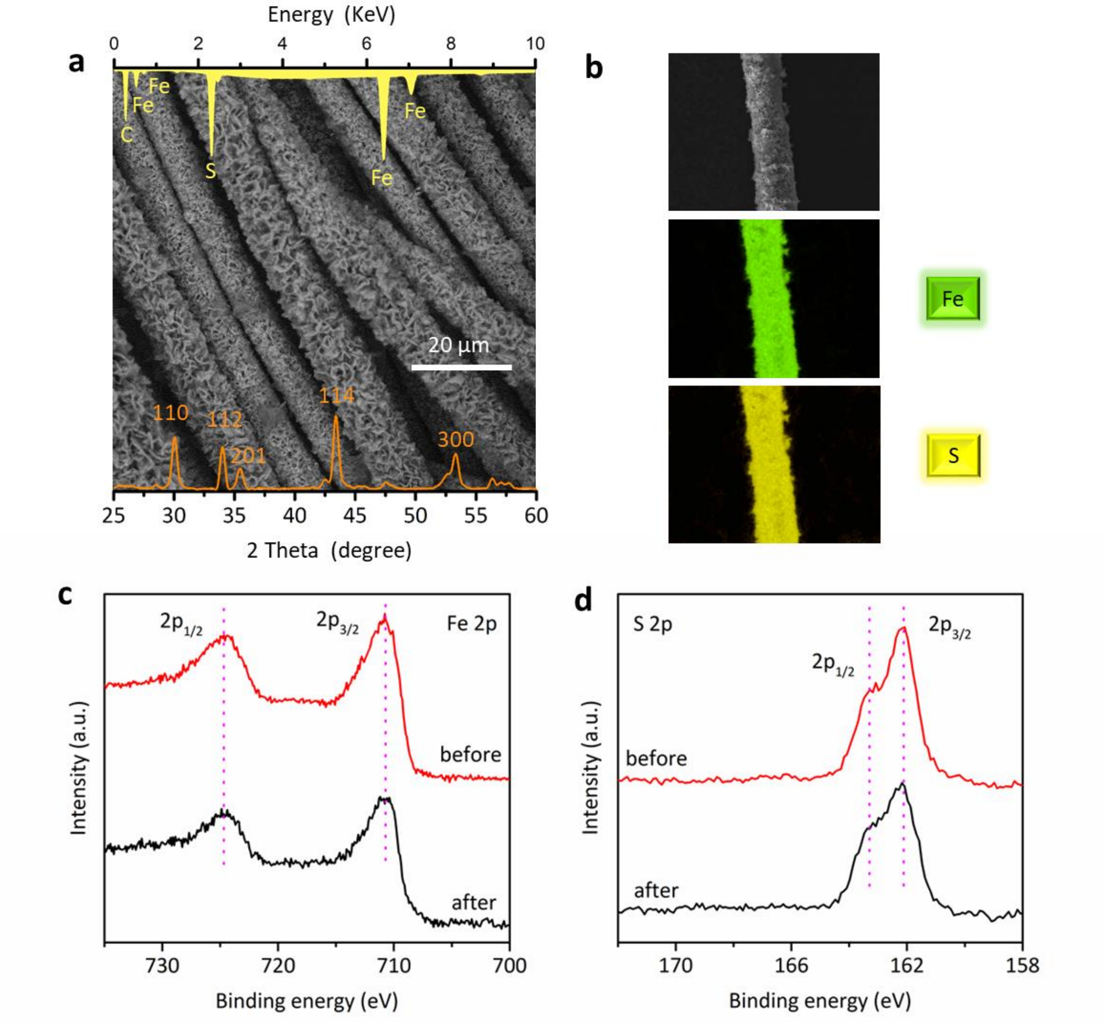

Supplementary Figure 20. (a) FE-SEM image, XRD pattern (shown as the orange overlaid at the bottom portion of the panel), EDX spectrum (shown as the yellow overlaid at the top portion of the panel), (b) EDS mapping and (c-d) XPS patterns of the FeS /CFC electrode under long-term stability testing.

By comparison, there are no discernible changes in the local morphology, crystal structure or chemical composition of FeS, which evidences the stability of the catalytic system. The XPS spectra of Fe 2p and S 2p for FeS nanosheets before and after catalysis were displayed in Supplementary Figures 20c-d, respectively. The spectra of Fe 2p for FeS nanosheets exhibit two peaks at 710.6 and 724.8 eV, which

are assigned to the $2p_{3/2}$ and $2p_{1/2}$ core levels of Fe^{2+}, respectively. Different sulfur species can be identified by the S 2p spectra in Supplementary Figure 20d. It is generally known that the major peaks at 162.1 and 163.2 eV could be ascribed to the disulfide ions (S^{2-}). The XPS spectra of S 2p before and after catalysis remain approximately unchanged, and no new XPS peaks corresponding to other sulfate moieties appear in our sample.

Supplementary Tables

Supplementary Table 1. HER parameters on FeS/CFC catalyst under different conditions.

Sample	Onset potential (mV vs. RHE) [a]	Over potential (mV vs. RHE) [b]	Tafel slope (mV per decade)
323 K Light	94	142	36.9
323 K Dark	101	199	51.8
298 K Light	102	192	51.4
298 K Dark	116	222	61.1

a. Onset potential is for achieving 0.5 mA cm^{-2}; **b**. over potential is for achieving 10 mA cm^{-2}.

Supplementary Table 2. The comparison of HER performance between FeS/CFC and previously reported Fe-based electrocatalysts.

Catalyst	Electrolyte solution	Overpotential (mV vs. RHE)	Exchange current density (j_0, μA cm^{-2})	Tafel slope (mV per decade)	Stability test	Reference
FeS/CFC	0.1 M KOH	142	3.1	36.9	100 h	This work
FeS_2/CoS_2	1.0 M KOH	78.2	–	44	80 h	Small, 2018, 14, 1801070
A-FeNiS	0.5 M H_2SO_4	105	2.2	40	40 h	J. Am. Chem. Soc. 2015, 137, 11900
$NiFe/NiCo_2O_4/NF$	1.0 M KOH	105	470	88	10 h	Adv. Funct. Mater. 2016, 26, 3515
Ni-Fe/NC	1.0 M KOH	219	–	110	1200 s	ACS Catal. 2016, 6, 580
Meso-FeS_2	0.1 M KOH	96	630	78	24 h	J. Am. Chem. Soc. 2017, 139, 13604
pyrite FeS_2 film	0.5 M H_2SO_4	270	–	62.5	4000 s	Energy Environ. Sci., 2013, 6, 3553
Pyrite FeS_2/C	0.1 M phosphate buffer	557	–	204	120 h	ACS Catal. 2016, 6, 2626
Ni Fe LDH-NS DG10	1.0 M KOH	300	–	110	20000 s	Adv. Mater. 2017, 29, 1700017
$FeS_2@MoS_2$/rGO	0.5M H_2SO_4	123	17.5	38.4	8 h	Chem. Commun., 2016, 52, 11795
FeS|P	0.5M H_2SO_4	160	–	44	2 h	ACS Catal. 2017, 7, 4026
2-cycle $NiFeO_x$/CFP	1.0 M KOH	88	–	150	100 h	Nature Comm. 2015, 6, 7261
FeS	1.0 M Phosphate buffer	350	0.66	150	120 h	ACS Catal. 2014, 4, 681
FeCoNi-HNT	1.0 M KOH	58	–	37.5	100 h	Nature Comm. 2018, 9, 2452
$(Fe_{0.48}Co_{0.52})S_2$	0.5 M H_2SO_4	196	0.959	47.5	20 h	J. Phys. Chem. C 2014, 118, 21347.
Fe-CoP/Ti	1.0 M KOH	78	–	75	20 h	Adv. Mater. 2017, 29, 1602441
Fe-N-C	0.5 M H_2SO_4	130	27	89	5 h	Adv. Energy Mater. 2018, 8, 1701345
Ni–Fe–P porous nanorods	1.0 M KOH	79	–	92.6	24 h	J. Mater. Chem. A, 2017, 5, 2496
$Co_{0.6}Fe_{0.4}P$/CNT	0.5 M H_2SO_4	67	–	57	24 h	Adv. Funct. Mater. 2017, 27, 1606635
FeP NAS/CC	1.0 M KOH	218	–	146	20 h	ACS Catal. 2014, 4, 4065

This table indicates that our synthesized FeS catalysts have the lowest Tafel slope and the excellent stability, and good catalytic activity. The differences in the performance parameters can be attributed to different nanostructures and substrates of various catalysts.

Supplementary Table 3. Comparison of HER catalytic parameters under different conditions.

Sample	Rs (Ω)	Rct (Ω)
323 K Light	1.15	13.12
323 K Dark	1.17	30.62
298 K Light	1.18	35.94
298 K Dark	1.21	41.51

The Rct value at 298 K is slightly reduced from 41.51 to 35.94 Ω due to NIR light irradiation, but the value at 323 K decreases nearly by half.

Supplementary Notes

Supplementary Note 1. The analysis about phase transition by MÖssbauer spectra and DFT.

To provide more substantial experimental evidences, ^{57}Fe MÖssbauer spectra were acquired from FeS sample with and without NIR light irradiation, as shown in in Supplementary Figure 10a. The schematic illustration of nuclear Zeeman splitting in in Supplementary Figure 10b indicates that hyperfine interaction induced by electric quadrupole and magnetic dipole leads to six typical resonance absorption, consistent with our expected MÖssbauer spectra. The obtained values of the isomer shift (IS= 0.83) and quadrupole splitting (QS=-0.15) without NIR light irradiation are comparable to previous data in the literature [3,4]. When the NIR light is introduced, the more symmetrical spectral fingerprint and the changed IS (0.79) and QS (-0.12) values are induced by reconfiguration of charge density accompanied by structural transformation. The analysis about atomic density of state (DOS) confirms that the highest occupied orbit is not composed by p-states of S atoms but d-states of Fe atoms (Supplementary Figure 7). And the corresponding electronic structure transition from semiconductor to metal phase will lead to an effective electron wave function rearrangement of d orbits, finally affecting electron density of s orbit nearby nucleus. By comparing the electron localization functions of two phases in Supplementary Figures 10c-d, we also found that the electrons have smaller tendency to locate around pristine atoms in metallic $P6_3/mmc$ phase (Supplementary Figures 10d) than those in semiconductor $P\bar{6}2_c$ phase (Supplementary Figures 10c), which can be used to explain the change in IS and QS value induced by phase transition. The changes in

both MÖssbauer spectra and corresponding analysis about electronic structure confirm that the phase transition can occur in our sample, which is quite in agreement with our conclusion associated with Raman spectra, photo-electricity response and comprehensive theoretical analysis.

Supplementary Methods

Chemicals and Reagents

Ferrous chloride, thiourea (toxic in contact with skin and if swallowed), and ethylene glycol were bought from Alfa Aesar. Sodium citrate, sulfuric acid, acetone (toxic by inhalation, in contact with skin and if swallowed), potassium hydroxide, and ethanol were obtained from Aladdin Industrial Corporation. The deionized water used throughout all experiments was doubly distilled water from a Millipore system. Carbon fiber clothes (CFC) were purchased from Phychemi (Hong Kong) Company Limited. All gases were purchased from Shangyuan industrial gas plant, China. All chemicals were of analytical grade and used as received without further purification.

Hydrogen adsorption energy and free energy calculation

The hydrogen adsorption free energy, ΔG_H, was determined in the same way as in previous studies[5]. The adsorption energy is defined as

$$\Delta E_H = E[FeS+H] - E[FeS] - ½\,E(H_2) \tag{19}$$

where [FeS+H] refers to hydrogen adsorbed on the FeS (100) surface, E[FeS] refers to a clean FeS surface, and H_2 refers to gas phase hydrogen molecule. The hydrogen adsorption free energy was calculated at zero potential as

$$\Delta G_H = \Delta E_H + \Delta E_{ZPE} - T\Delta S \tag{20}$$

where ΔE_H is the hydrogen adsorption energy, ΔE_{ZPE} is the difference in zero point energy, T is the temperature (300 K) and ΔS is the difference in entropy between H adsorbed and that in the gas phase, at 101325 Pa. A normal mode analysis was used to

determine the vibrational frequencies of the adsorbed species, which was used to determine the zero point energy correction and the entropy. The adsorption is too strong if ΔG_H is very negative or too weak if ΔG_H is very positive.

Electric near-field simulation

The electromagnetic simulation was performed using a Finite Element Method (FEM). In addition, the refractive index of water was set to be dispersive. All the physical domains were surrounded by perfectly matched layers (PML) to absorb all the outgoing waves, thus minimizing any reflection. Plane waves were used as excitation sources in all simulations along the Z axis. For better clarification, the intensity of electrical field distribution was visualized in a square scale.

Maxwell's equations were rigorously solved by three-dimensional (3D) finite-element method. The incident, internal and scattered electromagnetic fields of nanosheet can be described by introducing a series of cuboid harmonics. The absorption coefficient of the light wave was related to the polarization, wavelength and incident angle of the light. According to the Fresnel formula, the relationship between the absorption coefficient of the medium for TE polarization (s wave) and TM polarization (p wave) and the wavelength and incident angle can be calculated separately:

$$R_{\mathrm{TE}} = \frac{\sin^2(\theta - \phi)}{\sin^2(\theta + \phi)}, \tag{21}$$

$$T_{\mathrm{TE}} = \frac{n_2 \cos\phi}{n_1 \cos\theta} \cdot \frac{4\sin^2\phi\cos^2\theta}{\sin^2(\theta + \phi)}, \tag{22}$$

$$R_{\mathrm{TM}} = \frac{\tan^2(\theta - \phi)}{\tan^2(\theta + \phi)}, \tag{23}$$

$$T_{\mathrm{TM}} = \frac{n_2 \cos\phi}{n_1 \cos\theta} \cdot \frac{4\sin^2\phi \cos^2\theta}{\sin^2(\theta+\phi)\cos^2(\theta-\phi)}. \quad (24)$$

R_{TE}, T_{TE}, R_{TM} and T_{TM} are the reflection and transmission coefficients of s wave and p wave, respectively. n_1 and n_2 are the refractive index of the incident and outgoing regions. θ and ϕ, the incident angle and refraction angle satisfy the law of refraction.

Supplementary References

1. Li, H. Y. *et al.* Systematic design of superaerophobic nanotube array electrode comprised of transition-metalsulfides for overall water splitting. *Nat. Commun.* **9**, 2452 (2018).
2. Kibsgaard, J. & Jaramillo, T. F. Molybdenum phosphosulfide: an active, acidstable, earth-abundant catalyst for the hydrogen evolution reaction. *Angew. Chem. Int. Ed.* **53**, 14433–14437 (2014).
3. Giovanni，C. D. *et al.* Bioinspired iron sulfide nanoparticles for cheap and long-lived electrocatalytic molecular hydrogen evolution in neutral water. *ACS Catal.* **4**, 681−687 (2014).
4. Oshtrakh, M. I. *et al.* Iron sulfide (troilite) inclusion extracted from Sikhote-Alin iron meteorite: Composition, structure and magnetic properties. Mater. Chem. Phys., **174**, 100-111 (2016).
5. Acevedo, M. C. *et al.* Efficient hydrogen evolution catalysis using ternary pyrite-type cobalt phosphosulphide. *Nat. Mater.* **14**, 1245-1251 (2015).
